# A cluster analysis of operating room nurses’ profiles based on their clinical decision-making for pressure injury prevention

**DOI:** 10.3389/fpubh.2025.1663952

**Published:** 2026-01-12

**Authors:** Zhen-Shan Guo, Xiu-Mei Wang, Hai-Long Fan, Ling Wei, Xue Zhang, Yue Guo, Yan-Bin Niu, Yan-Jie Li

**Affiliations:** 1 Department of Central Surgery Department, Shanxi Bethune Hospital, Shanxi Academy of Medical Sciences, Tongji Shanxi Hospital, Third Hospital of Shanxi Medical University, Taiyuan, China; 2 Department of Disinfecting Supply Division, Shanxi Bethune Hospital, Shanxi Academy of Medical Sciences, Tongji Shanxi Hospital, Third Hospital of Shanxi Medical University, Taiyuan, China

**Keywords:** clinical competence, cluster analysis, decision making, operating room nursing, pressure injury

## Abstract

**Objective:**

To comprehend the potential categories of operating room nurses based on their clinical decision-making capability for pressure injuries and to analyze the characteristics of nurses falling under the various categories.

**Methods:**

A cross-sectional survey was conducted from June to August 2022 using convenient sampling, with 469 operating room nurses from 12 tertiary hospitals in Shanxi, Guangxi, Sichuan, Guizhou, Yunnan, Xinjiang, and Chongqing as the participants. The General Information Questionnaire, Clinical Decision-Making in Nursing Scale, Chinese Critical Thinking Disposition Inventory, Pressure Injury Management Self-Efficacy Scale, and Transformational Leadership Questionnaire were used in the survey. Clinical decisions taken by nurses in case of pressure injuries were analyzed using profiles, and the influencing factors of different categories were analyzed using multivariate logistic regression.

**Results:**

The clinical decision-making of operating room nurses for pressure injuries can be divided into 3 profile groups: poor clinical decision-making group (40.3%), moderate clinical decision-making group (26.0%), and good clinical decision-making group (33.7%). The influencing factors of clinical decision-making capability for pressure injuries in operating room nurses were critical thinking ability, self-efficacy, transformational leadership, and educational background (all *p* < 0.05).

**Conclusion:**

We developed three clinical decision-making profiles of operating room nurses for pressure injuries. Nursing managers can implement targeted intervention strategies based on the characteristics of individual nurses to improve clinical decision-making.

## 1 Introduction

Operating room nurses play a crucial role in the management of pressure injuries, typically functioning in two distinct roles: scrub nurses and circulating nurses. Scrub nurses are primarily responsible for sterile technique management, instrument handling, and direct assistance to surgeons during procedures, while circulating nurses coordinate patient positioning, monitor vital signs, manage documentation, and ensure overall operating room safety and efficiency. Both roles require distinct yet complementary decision-making capabilities in pressure injury prevention. These role-specific responsibilities shape distinct decision-making foci: scrub nurses must instantaneously decide on instrument selection and sterility maintenance, whereas circulating nurses integrate patient-risk data to decide on positioning devices and skin-protection measures.

Pressure injuries (PIs) represent a significant challenge in perioperative nursing care, with an incidence rate as high as 5 to 53% among surgical patients. Operating room (OR) nurses play a crucial role in the management of pressure injuries (1). Clinical decision-making ability refers to the capacity of healthcare professionals to make optimal clinical choices based on their professional knowledge and clinical experience through systematic assessment and analytical judgment. For OR nurses, this ability is manifested in accurately identifying risk factors for PIs, selecting appropriate preventive measures, and timely adjusting nursing plans, all of which are critical components of PI management. The unique environment of the operating room (e.g., prolonged immobilization, effects of anesthesia, and specific surgical positions) significantly increases the risk of PIs in patients. The consequences of PIs are not limited to patient suffering; they also extend to increased healthcare costs and prolonged hospital stays, underscoring the urgency of effective prevention strategies (2).

Research has shown that the clinical decision-making ability of nurses directly impacts the effectiveness of PI prevention, primarily through three mechanisms: First, by identifying high-risk patients through professional assessment [e.g., those with surgical duration 3h or abnormal body mass index (BMI)] (3); second, by promptly detecting early signs of injury through clinical observation (e.g., non-blanchable erythema); and third, by effectively allocating resources in a multitasking environment to ensure the implementation of preventive measures. Notably, even with the implementation of standardized protocols, the incidence of PIs remains at 12 to 18% (4). This persistent high rate highlights the limitations of a one-size-fits-all protocol-based approach and strongly suggests that enhancing the individual clinical decision-making abilities of nurses is a critical, yet underexplored, avenue for improving patient outcomes (5).

Decision-making is a critical non-technical skill, refers to the capacity of healthcare professionals to make optimal clinical choices based on their professional knowledge and clinical experience through systematic assessment and analytical judgment (6).and its study in the context of OR nursing has gained increasing attention. Former studies on OR nurses’ non-technical skills have extensively explored areas such as teamwork, communication, and situational awareness, all of which are vital for patient safety (7). However, a specific focus on the nuanced nature of clinical decision-making, particularly in the domain of PI prevention, remains less developed (8). Existing research often treats decision-making as a monolithic construct, failing to capture the potential heterogeneity among nurses. This oversight limits the ability of nurse managers to design targeted training and interventions, as the underlying profiles of decision-makers are not well understood. There is a clear necessity to move beyond average scores and investigate the distinct subgroups that may exist within the OR nursing population regarding their decision-making capabilities (9, 10).

To address this gap, this study employs latent profile analysis, a person-centered statistical method, to identify distinct subgroups of OR nurses based on their clinical decision-making capabilities for pressure injuries. This approach allows for a more granular understanding of how these capabilities cluster among individuals, rather than treating them as isolated variables. The objectives of this study are: (1) to identify and characterize the potential profiles of OR nurses based on their clinical decision-making for PI management, and (2) to analyze the factors that influence membership in these different profiles. By achieving these objectives, we aim to provide a robust empirical foundation for developing tailored, evidence-based intervention strategies. Ultimately, this research seeks to empower nursing managers to implement targeted capacity-building programs that address the specific needs of different nurse profiles, thereby enhancing the overall quality of PI prevention and improving surgical patient safety.

## 2 Participants and methods

### 2.1 Study design

A single-time, cross-sectional descriptive-analytic design was used with the individual nurse as the unit of analysis.

The study proceeded in two stages:

*Descriptive stage*: Between June and August 2022, convenience sampling was used to recruit operating-room nurses from 12 tertiary hospitals in seven Chinese provinces. Data were collected through five validated Chinese-language questionnaires (demographic form, Clinical Decision-Making in Nursing Scale, Critical Thinking Disposition Inventory, Pressure-Injury Management Self-Efficacy Scale, and Transformational Leadership Questionnaire).

*Analytic stage*: Latent Profile Analysis (LPA) was first performed on the total and four sub-scale scores of the CDMNS to identify the optimal number of latent profiles of pressure-injury clinical decision-making ability. Profile membership (three classes: Poor, Moderate, Good) was then used as the outcome in a multinomial logistic regression examining the effects of critical-thinking disposition, self-efficacy, transformational leadership, and educational background on profile allocation. The regression followed the 10-events-per-variable rule for seven predictors.

Data were collected via the Wenjuanxing platform. LPA was run in Mplus 7.4; remaining analyses were conducted in SPSS 19.0. The study was approved by the Hospital Ethics Committee (approval No. 2021089) and all participants gave electronic informed consent.

### 2.2 Participants

A cross-sectional survey was conducted from June to August 2022. To improve representativeness, we employed a two-stage stratified cluster sampling approach. First, seven provinces/municipalities (Shanxi, Guangxi, Sichuan, Guizhou, Yunnan, Xinjiang and Chongqing) were purposely selected to cover different geographical regions of China. Second, in each selected region we randomly chose one or two tertiary hospital and then recruited all eligible operating-room nurses from the surgical departments of these hospitals (cluster unit). Inclusion criteria: (1) Nurses who have worked in an operating room for 5 years or more; (2) Nurses who care for patients undergoing surgery with a high risk of pressure injuries. Exclusion criteria: (1) Nurses who are absent for various reasons; (2) Nurses who are pursuing further education.

The sample-size calculation was tailored to the multinomial logistic regression used in the analytic stage. The most conservative rule-of-thumb for multinomial models recommends ≥ 10 outcome events per parameter for the least frequent category (11). Pilot data (*n* = 60) showed the expected proportions of the three latent profiles to be 35% “poor,” 45% “moderate” and 20% “good.” Treating the smallest group (“good,” 20%) as the reference category and planning to enter seven predictors (*k* = 7) implied a minimum of (10 × 7)/0.20 = 350 cases. After allowing for an anticipated 20% invalid response rate, we set the target sample at ≥ 420 participants. Ultimately, a total of 503 questionnaires were collected, and 469 were valid, for an effective recovery rate of 93.24%, which exceeded the required sample size.

### 2.3 Methods

#### 2.3.1 Research tools

##### 2.3.1.1 General information questionnaire

The general information questionnaire is self-designed and includes demographic data such as gender, age, education, marital status, years of working, title, and personnel status.

##### 2.3.1.2 Clinical decision-making in nursing scale (CDMNS)

The awareness of clinical decision-making of nurses was measured using the Chinese version of the CDMNS modified by Guo (12), with the Cronbach’s *α* coefficient of the scale at 0.850. The confirmatory factor analysis (CFA) showed good structural validity with *χ*^2^/df = 2.45, CFI = 0.92, TLI = 0.90, and RMSEA = 0.06. The test–retest reliability over a 2-week interval was 0.78, and the split-half reliability was 0.82. These psychometric properties were established in a sample of 469 operating room nurses. The scale includes 40 items divided into 4 dimensions: finding solutions to problems (10 items), determining the goal of problem solving (10 items), repeatedly evaluating the effect of implementation (10 items), and searching for relevant information again (10 items). The Likert 5-level scoring method was used, and each entry was assigned 1–5 points ranging from “never” to “always,” respectively, with a total score of 40–200 points. The higher the score, the greater the nurse’s clinical decision-making ability in nursing (13, 14).

##### 2.3.1.3 Chinese critical thinking disposition inventory (CTDI-CV)

The nurses’ critical thinking ability was measured using the Chinese version of the CTDI-CV scale modified by Peng et al. (15), with the Cronbach’s *α* coefficient of the scale at 0.90. The CFA results demonstrated adequate construct validity (*χ*^2^/df = 2.89, CFI = 0.91, TLI = 0.89, RMSEA = 0.07). The test–retest reliability was 0.85, and the split-half reliability was 0.88. The validation study included 469 operating room nurses in the current sample. Truth-seeking, open-mindedness, analytical ability, systematization ability, self-confidence, intellectual curiosity, and cognitive maturity were the 7 dimensions of the scale. Each dimension has 10 items, for a total of 70 items. The Likert 6-level scoring method was used, with 6 to 1 points scored for each item, ranging from “strongly agreed” to “strongly disagreed,” respectively, with a total score of 70–420 points (16). A score of more than 280 points indicated a positive tendency toward critical thinking, and a score of more than 350 points indicated a strong positive tendency toward critical thinking (17).

##### 2.3.1.4 Pressure injury management self-efficacy scale

The self-efficacy of nurses in pressure injury management was measured using the pressure injury management self-efficacy scale Chinese version modified by Hou et al. (18), with the Cronbach’s *α* coefficient of the scale at 0.967. Excellent structural validity was confirmed through CFA (*χ*^2^/df = 1.98, CFI = 0.96, TLI = 0.95, RMSEA = 0.04). The test–retest reliability over 3 weeks was 0.91, and the split-half reliability was 0.93. These reliability and validity indices were based on the current sample of 469 operating room nurses. With a total of 10 items, the scale includes 4 dimensions: evaluation (2 items), planning (2 items), management (2 items), and decision-making (4 items). The Likert 5-level scoring method was used, and each entry received 1–5 points ranging from “cannot do” to “fully capable,” respectively, with a total score of 10–50 points. The higher the score, the greater the nurse’s self-efficacy in pressure injury management (19).

##### 2.3.1.5 Transformational leadership questionnaire (TLQ)

The TLQ developed by Li and Shi (20),was used to assess the degree of transformational leadership behavior of nursing supervisors as perceived by operating room nurses. Among nursing staff, the measured Cronbach’s *α* coefficient was 0.946 (21). The CFA confirmed good construct validity (*χ*^2^/df = 2.67, CFI = 0.93, TLI = 0.91, RMSEA = 0.05). The test–retest reliability was 0.89, and the split-half reliability was 0.92. All psychometric properties were evaluated in the present sample of 469 operating room nurses. With a total of 26 items, the scale includes 4 dimensions: virtue (8 items), leadership charisma (6 items), personalized care (6 items), and vision motivation (6 items). The Likert 5-level scoring method was used, and each entry received 1–5 points from “strongly disagreed” to “strongly agreed,” respectively, for a total score of 26–130 points (22). The higher the score, the greater the degree of transformational leadership behavior of the nursing supervisor perceived by operating room nurses (23).

#### 2.3.2 Investigation method

The study was conducted using the WeChat group of the Western Nursing Society Research Center’s operating room anesthesia professional committee. The questionnaire QR code and link were sent to the WeChat group by the researchers. The form was then distributed to the nurses, who were invited to fill it out.

Before the investigation, participants were informed of the purpose and significance of the investigation and the precautions. In addition, we pledged to protect personal privacy and data security. After the questionnaire collection was completed, the questionnaires were checked and verified individually by the researchers. Exclusion criteria for the questionnaire were (1) incorrect logic or answers in a fixed pattern; (2) an incomplete questionnaire (20% or more).

#### 2.3.3 Statistical methods

The SPSS19.0 software was used for data analysis. The measurement data are expressed as mean ± standard deviation, and the between-group comparison was performed using the analysis of variance or the Kruskal-Wallis H test. The counting data are expressed as a frequency and percentage, and the chi-squared test was used to compare groups. Multivariate logistic regression was used to analyze the influencing factors of clinical decision-making capability for pressure injury by nurses with different profiles, and the test level *α* was 0.05. Model-fit criteria are described in Section 2.1. If the *p* value was less than 0.05, it indicated that the k classification models fit better than the k-1 models (24).

## 3 Results

### 3.1 General data

The average age of the operating room nurses was (34.56 ± 2.36) years, and the rest of the general data are shown in Table 1.

**Table 1:** Univariate analysis of operating room nurse profiles based on clinical decision-making capability for pressure injury.

Variables	Class 1 (*n* = 189)	Class 2 (*n* = 122)	Class 3 (*n* = 158)	F/*χ*^2^ value	*p* value
Critical thinking (score, *x̄* ± *s*)	270.41 ± 23.46^b^	282.86 ± 25.67^a^	296.61 ± 27.24^ab^	45.945	<0.001
Self-efficacy (score, *x̄* ± *s*)	22.67 ± 4.21^b^	27.51 ± 5.68^a^	34.76 ± 6.71^ab^	205.54	<0.001
Transformational leadership (score, *x̄* ± *s*)	70.24 ± 8.12	75.86 ± 8.64	81.81 ± 8.89^ab^	79.405	<0.001
Gender				2.369	0.687
Male	22 (11.64)	14 (11.48)	18 (11.39)		
Female	167 (88.36)	108 (88.52)	140 (88.61)		
Age (years)				3.578	0.621
29 ~ 35	96 (50.79)	63 (51.64)	82 (51.90)		
36 ~ 40	56 (29.63)	35 (28.69)	46 (29.11)		
41 ~ 46	37 (19.58)	24 (19.67)	30 (18.99)		
Educational background				23.698	<0.001
Junior college level or lower	87 (46.03)	36 (29.51)	29 (18.35)		
Bachelor’s degree	60 (31.75)	41 (33.61)	50 (31.65)		
Master’s degree or higher	42 (22.22)	45 (36.88)	79 (50.00)		
Marital status				2.623	0.665
Unmarried	56 (29.63)	37 (30.33)	49 (31.01)		
Married	69 (36.51)	46 (37.70)	58 (36.71)		
Divorced or others	64 (33.86)	64 (33.86)	51 (32.28)		
Years of working (years)				1.674	0.869
8 ~ 12	98 (51.85)	63 (51.64)	82 (51.90)		
13 ~ 15	57 (30.16)	36 (29.51)	47 (29.75)		
≥16	34 (17.99)	23 (18.85)	29 (18.35)		
Title				1.823	0.762
Nurse practitioner	99 (52.38)	64 (52.46)	83 (52.53)		
Supervisor nurse	56 (29.63)	37 (30.33)	48 (30.38)		
Associate chief nurse or higher	34 (17.99)	21 (17.21)	27 (17.09)		
Personnel status				2.046	0.573
Officially permanent employees	81 (42.86)	51 (41.80)	65 (41.14)		
Unofficially permanent employees	108 (57.14)	71 (58.20)	93 (58.86)		

^a^*p* < 0.05, when compared to Class 1. ^b^*p* < 0.05, when compared to Class 2.

### 3.2 Analysis of operating room nurse profiles based on clinical decision-making capability for pressure injury

The average scores of the operating room nurses in clinical decision-making capability for pressure injury were (2.21 ± 0.48) points. The 40 items of the CDMNS were used as explicit indicators, and 1–4 potential profile models were selected (Table 2). Class 3 had lower LL, AIC, BIC, and aBIC values than Class 1 and Class 2, and the difference in LMR (*p* < 0.05) and BLRT (*p* < 0.05) was statistically significant. However, though the LL, AIC, BIC, and aBIC values of Class 4 were lower than those of Class 3, there was no statistically significant difference in LMR (*p* = 0.413). Therefore, 3 potential profile classifications were selected for this study.

**Table 2:** Operating room nurse profile analysis indicators for clinical decision-making capability for pressure injury (*n* = 469).

Classification model	LL	AIC	BIC	aBIC	Entropy	LMR (P)	BLRT (P)	Classification Percentage (%)
Class 1	−9294.534	18646.863	18687.862	18742.761	–	–	–	–
Class 2	−7685.526	15368.238	15722.697	15675.637	0.974	<0.001	<0.001	39.7/64.6
Class 3	−7313.671	14786.698	15035.230	14837.148	0.936	<0.013	<0.001	26.8/34.8/44.2
Class 4	−7055.786	14345.637	14613.479	14468.368	0.958	0.451	<0.001	24.5/39.1/4.8

### 3.3 Identification of operating room nurse profiles based on clinical decision-making capability for pressure injury

**Figure 1:**
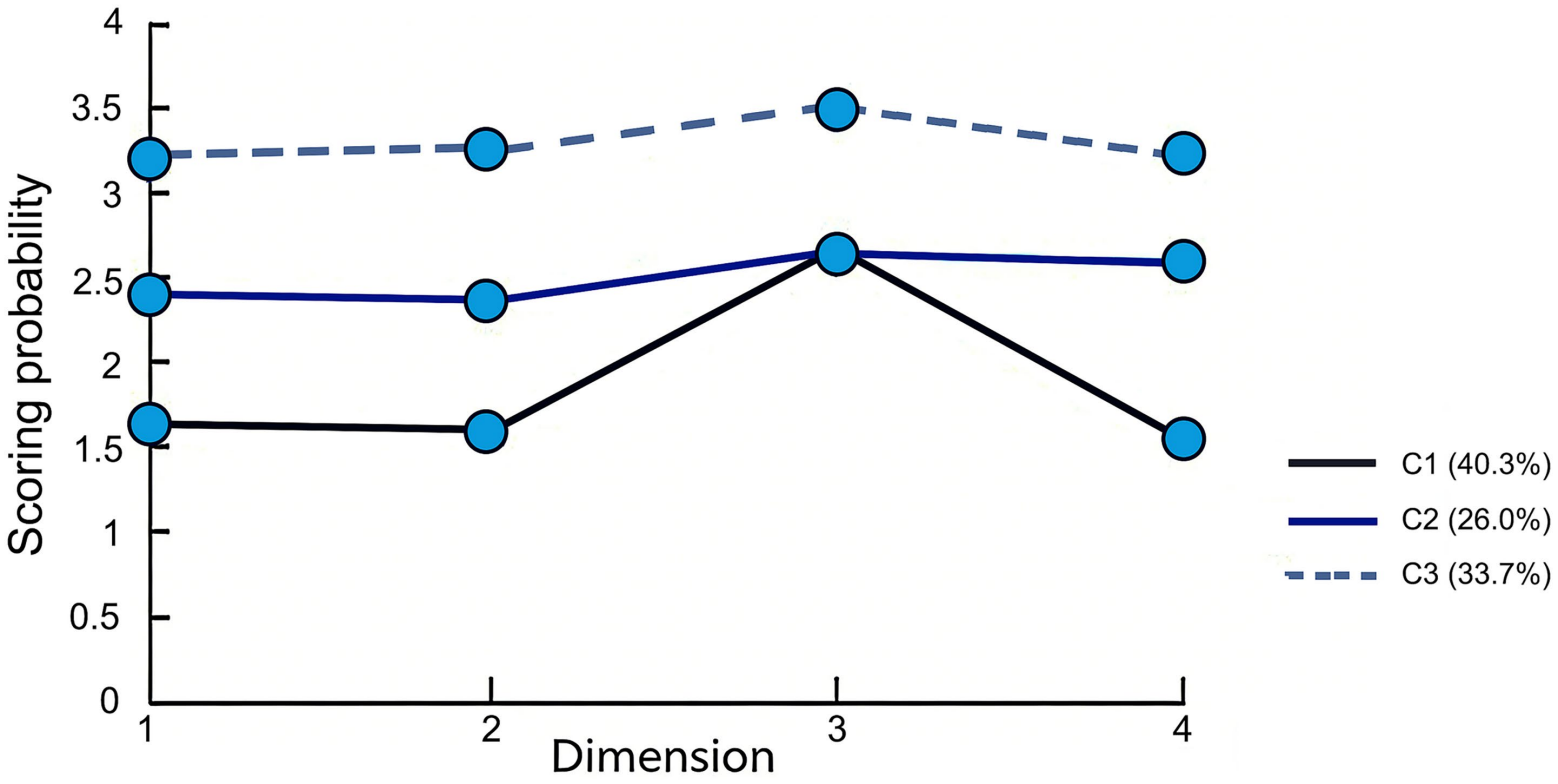
Score probability of the three operating room nurse profiles based on clinical decision-making capability for pressure injury under four dimensions.

Figure 1 depicts the score probabilities of three operating room nurse profiles for clinical decision-making capability for pressure injury under four dimensions. The Class 1 category, which is rated as the “poor clinical decision-making group,” scored the lowest in all dimensions, accounting for 40.3%. The proportion of the Class 2 category was 26.0%, and the scores in all dimensions were higher than those of Class 1 but lower than those of Class 3. Therefore, the Class 2 category was named “moderate clinical decision-making group.” The Class 3 category had the highest proportion (33.7%) in all dimensions. Therefore, the Class 3 category was named the “good clinical decision-making group.”

### 3.4 Univariate analysis of operating room nurse profiles based on clinical decision-making capability for pressure injury

Critical thinking, self-efficacy, transformational leadership, and educational background were statistically significant in the three profile categories for clinical decision-making capability for pressure injury in operating room nurses (*p* < 0.05) (Table 1).

### 3.5 Multivariate analysis of operating room nurse profiles based on clinical decision-making capability for pressure injury

The multivariate logistic regression analysis was conducted with three profile categories as dependent variables; the variables with statistically significant difference in the analysis of different characteristics of the three profile categories were set as independent variables. The following independent variables were assigned: junior college degree or below = 1, bachelor = 2, master’s degree or above = 3. The scores of critical thinking, self-efficacy, and transformational leadership were assigned as the original values. The results revealed that an educational background of junior college or below was a risk factor for clinical decision-making capability for pressure injury by operating room nurses (OR = 3.638, *p* < 0.001). The higher the critical thinking score, the better the clinical decision-making of operating room nurses on pressure injury (OR = 0.981, *p* < 0.001). The higher the self-efficacy score, the better the clinical decision-making of operating room nurses on pressure injury (OR = 0.990, *p* < 0.001). The higher the transformational leadership score, the greater the clinical decision-making of operating room nurses on pressure injury (OR = 0.987, *p* < 0.001) (Table 3).

**Table 3:** Multivariate analysis of operating room nurse profiles based on clinical decision-making for pressure injury.

Items	Regression coefficient	Standard error	Wald *χ*^2^	*p*	OR	95% confidence interval
Constant	7.976	1.864	21.784	<0.001		
Educational background	1.696	0.341	14.689	<0.001	3.638	[1.874, 6.763]
Critical thinking	0.052	0.031	15.471	<0.001	0.981	[0.996, 0.952]
Self-efficacy	0.067	0.063	16.041	<0.001	0.990	[0.924, 0.958]
Transformational leadership	0.079	0.064	15.542	<0.001	0.987	[0.963, 0.989]

## 4 Discussion

### 4.1 Group heterogeneity among operating room nurses in clinical decision-making capability for pressure injury

This study provides new insights into the clinical decision-making abilities of operating room nurses in the management of pressure injuries (PI) through latent profile analysis. Using latent profile analysis, we identified three distinct patterns of clinical decision-making for pressure-injury prevention among 469 operating-room nurses: poor (40%), moderate (26%) and good (34%). The finding that two-thirds of staff cluster in the poor-to-moderate profiles mirrors recent European data (1) (38% poor, 29% moderate, 33% good), which also aligns with recent research indicating that protocol-based education alone is insufficient to foster the complex cognitive processes required for dynamic clinical judgment (1, 2, 6, 25). Nurses in the poor profile are at highest risk of failing to recognise early pressure-injury signs or to prioritise interventions during prolonged surgery; simulation-based training with feedback is therefore urgently indicated for this subgroup (26), whereas the moderate group may benefit from complex scenario rehearsal only. This person-centered approach to education is increasingly recognized as a best practice in healthcare professional development (5).

### 4.2 Factors that may influence the clinical decision-making capability of operating room nurses for pressure injury

#### 4.2.1 Highly educated operating room nurses make excellent clinical decisions regarding pressure injury

According to the findings of this study, operating room nurses with a master’s degree or above had a higher clinical decision-making level for pressure injury (OR = 3.638), which is consistent with the results of previous research (23), suggesting that the high-stakes, time-constrained OR environment amplifies the educational gradient. One plausible mechanism is that university curricula expose nurses to pathophysiology of pressure injuries beyond the protocol level, enabling rapid risk stratification when surgical duration exceeds 2.5 h, a cutoff already shown in previous study to precede most PI events (3). These nurses with higher education generally possess more resources to perform a range of additional roles. It suggests that higher education not only imparts specialized knowledge but also cultivates the critical thinking and analytical skills essential for high-level clinical judgment (27).

#### 4.2.2 Operating room nurses with strong critical thinking skills make sound clinical decisions about pressure injuries

The results of this study indicated that higher critical thinking ability was associated with higher clinical decision-making on pressure injury in operating room nurses. Every 10-point rise on the CTDI-CV reduced the odds of belonging to the poor-decision profile by 19% (OR = 0.981). This dose–response pattern aligns with the 15% reduction observed by Hsu et al. among maternal-nursing students (28), but the effect size is smaller, probably because OR decisions must be made under anesthesia-induced information gaps. We argue that nurses with high critical thinking ability are flexible in their thinking, actively respond to situations and can assess and intervene in case of pressure injuries. This highlights the need for educational curricula and in-service training programs to explicitly integrate critical thinking frameworks, such as problem-based learning and case-based reasoning, rather than relying solely on didactic knowledge transfer (28, 29). Case analysis, role playing, group discussions, and so on. should be used to improve critical thinking ability and improve the capabilities of nurses in clinical decision-making on pressure injury.

#### 4.2.3 Operating room nurses with high self-efficacy possess high clinical decision-making abilities for pressure injuries

The results of this study revealed that self-efficacy influenced clinical decision-making capability of operating room nurses for pressure injury (*p* < 0.05). According to Notarnicola et al. (30), there are two methods for nursing clinical decision-making: pattern recognition and systematic analysis. Pattern recognition is primarily based on the accumulation of experience by nurses, whereas systematic analysis is primarily used to analyze complicated and difficult problems. Making a good assessment of pressure injury is critical for operating room nurses in terms of improving self-efficacy in managing pressure injury in surgical patients (31, 32). Tura et al. (33) developed a risk factor assessment scale for intraoperatively acquired pressure injury, for which multi-center applications and research were carried out in seven hospitals across the country. The use of such validated tools can provide objective data, which in turn can bolster a nurse’s confidence in their assessments and interventions, thereby creating a positive feedback loop that enhances both self-efficacy and decision-making quality (34).

#### 4.2.4 Operating room nurses with high levels of transformational leadership have high clinical decision-making on pressure injury

The results of this study showed that the more obvious the transformational leadership of the head nurse as perceived by the operating room nurses, the higher the clinical decision-making capability of the operating room nurses for pressure injury, which is consistent with the results of Collins et al. (23) and Wang et al. (24). The transformative leadership style not only makes nurses feel that their work tasks are important but also plays a role in guiding, motivating, directing, driving, and inspiring higher career expectations in nurses, and assisting nurses in enhancing clinical decision-making capability for pressure injury. This finding provides strong evidence for investing in leadership development programs for nurse managers (35, 36). By fostering transformational leadership behaviors (37), healthcare organizations can create a supportive and empowering climate that indirectly but powerfully improves clinical outcomes by enhancing the decision-making capacities of their nursing staff (38). It is suggested that nursing managers need to strengthen the training of head nurses in transformational leadership knowledge, skills, and leadership, and further improve the transformational leadership skills of head nurses.

Three limitations merit explicit mention. This study was a cross-sectional survey of operating room nurses in some tertiary hospitals in West China, which restricts generalisability to other health-care contexts. Although scales were validated, social-desirability tendencies may have inflated self-efficacy and leadership scores. In future studies, a large-sample survey shall be conducted to further investigate the influencing factors of clinical decision-making capabilities of operating room nurses for pressure injury. Longitudinal or experimental designs are required to confirm whether enhancing critical thinking or transformational leadership actually shifts nurses from poor to good clinical decision-making categories.

## 5 Conclusion

The clinical decision-making capability of operating room nurses for pressure injury can be classified into three groups: poor clinical decision-making group, moderate clinical decision-making group, and good clinical decision-making group. Critical thinking ability, self-efficacy, transformational leadership, and educational backgrounds of nurses differ across categories. Nursing managers should be skilled at identifying the characteristics of nurses in the poor clinical decision-making group and in assisting them in improving their clinical decision-making capability for pressure injury by implementing transformative leadership and creating a positive self-efficacy environment. This study advocates for a paradigm shift from generic, protocol-driven training to personalized, profile-based interventions. By understanding the distinct characteristics and needs of different nurse subgroups, administrators can more effectively allocate resources to foster a highly competent and resilient nursing workforce, ultimately leading to safer patient care.

## Data Availability

The original contributions presented in the study are included in the article/supplementary material, further inquiries can be directed to the corresponding authors.
